# Magnetic resonance – guided treatment of low-flow vascular malformations and the technologies to potentiate adoption

**DOI:** 10.3389/fmed.2024.1319046

**Published:** 2024-02-14

**Authors:** Christopher Ravi Bailey, Daniel Giraldo Herrera, Nicolas Neumeister, Clifford Rabbe Weiss

**Affiliations:** ^1^Department of Radiology and Radiological Science, School of Medicine, Johns Hopkins Medicine, Baltimore, MD, United States; ^2^Goucher College, Baltimore, MD, United States

**Keywords:** low flow vascular malformations, MRI guidance, interventional MRI, ablation, laser, cryoablation, sclerotherapy

## Abstract

Vascular malformations are congenital, non-neoplastic lesions that arise secondary to defects in angiogenesis. Vascular malformations are divided into high-flow (arteriovenous malformation) and low-flow (venous malformations and lymphatic malformations). Magnetic resonance imaging (MRI) is the standard for pre-and post-intervention assessments, while ultrasound (US), X-ray fluoroscopy and computed tomography (CT) are used for intra-procedural guidance. Sclerotherapy, an image-guided therapy that involves the injection of a sclerosant directly into the malformation, is typically the first-line therapy for treating low-flow vascular malformations. Sclerotherapy induces endothelial damage and necrosis/fibrosis with eventual involution of the malformation. Image-guided thermal therapies involve freezing or heating target tissue to induce cell death and necrosis. MRI is an alternative for intra-procedural guidance and monitoring during the treatment of vascular malformations. MR can provide dynamic, multiplanar imaging that delineates surrounding critical structures such as nerves and vasculature. Multiple studies have demonstrated that MR-guided treatment of vascular malformations is safe and effective. This review will detail (1) the use of MR for the classification and diagnosis of vascular malformations, (2) the current literature surrounding MR-guided treatment of vascular malformations, (3) a series of cases of MR-guided sclerotherapy and thermal ablation for the treatment of vascular malformations, and (4) a discussion of technologies that may potentiate interventional MRI adoption including high intensity focused ultrasound and guided laser ablation.

## Introduction to vascular anomalies

Vascular anomalies are benign tumors comprising a variety of defects in vasculogenesis. These anomalies are distinguished by the widely accepted International Society for the Study of Vascular Anomalies (ISSVA) classification system 2018 update (1, 2). Vascular malformations are categorized as simple: malformations that are characterized by a single vascular type; or combined: malformations that involve two or more vascular types. Vascular anomalies are also subdivided into low-flow and high-flow malformations. Simple low flow vascular malformations (LFVMs) include venous malformations (VMs) and lymphatic malformations (LMs). These lesions consist of disorganized mass-like venous and lymphatic channels that can arise in any part of the body and range in size from solitary lesions to diffuse masses. Common clinical manifestations of LFVMs include pain, bleeding, swelling, and disfigurement.

## Traditional diagnostic and treatment modalities

The gold standard for diagnosis and post-treatment monitoring of LFVMs is magnetic resonance imaging (MRI). MRI allows for detailed characterization of the lesion and can uncover associated lesions in the case of genetic or sporadic syndromes. Ultrasound (US), X-ray fluoroscopy, and computed tomography (CT) are commonly used for intra-procedural guidance; however, they may have some diagnostic utility in select cases. While ultrasound offers adequate soft tissue resolution and real-time imaging with no ionizing radiation, many lesions cannot be wholly visualized since resolution decreases with depth. Furthermore, LFVMs obscured by bone or air-filled tissue are not well visualized with US. Ultrasound is often used for guiding needle placement during sclerotherapy procedures and can also be used to monitor sclerosant delivery. Fluoroscopy provides the advantage of real-time intraprocedural imaging, specifically sclerosant delivery, flow, and outflow. However, fluoroscopy has poor tissue resolution relying on contrast media to opacify the vascular structures of the malformation. It also exposes the patient and operator to potentially significant doses of ionizing radiation, making this technique less viable for repeated treatments especially in pediatric patients (3). CT offers adequate soft tissue resolution and a wide anatomic field of view which is helpful for initial treatment planning; however, it does not offer the same degree of soft tissue resolution to make definitive diagnosis like MRI. Regarding intraprocedural navigation, CT often allows lesion targeting by landmarks alone which has the risk of poor accuracy and limited repeatability when targeting a lesion. Like fluoroscopy, CT involves a radiation exposure to the patient and the operator.

Traditionally, first line therapy for symptomatic lesions involves US, CT, or fluoroscopy guided sclerotherapy (4). In this approach, the vascular channels/cystic spaces of these lesions are accessed with needles under ultrasound guidance. Fluoroscopic digital subtraction angiography (DSA) is then used intraprocedural to assess flow, outflow, and guide sclerosant delivery while monitoring for thrombosis in real-time. Percutaneous interventions may be followed by surgical excision as part of a staged treatment plan for complex multiplanar lesions (4, 5). Though ultrasound and fluoroscopy are primarily used in the visualization and treatment of LFVMs, as stated above, not all malformations may be accessed with these modalities.

## Two-fold advantage of MRI-guided therapy

**Figure 1:**
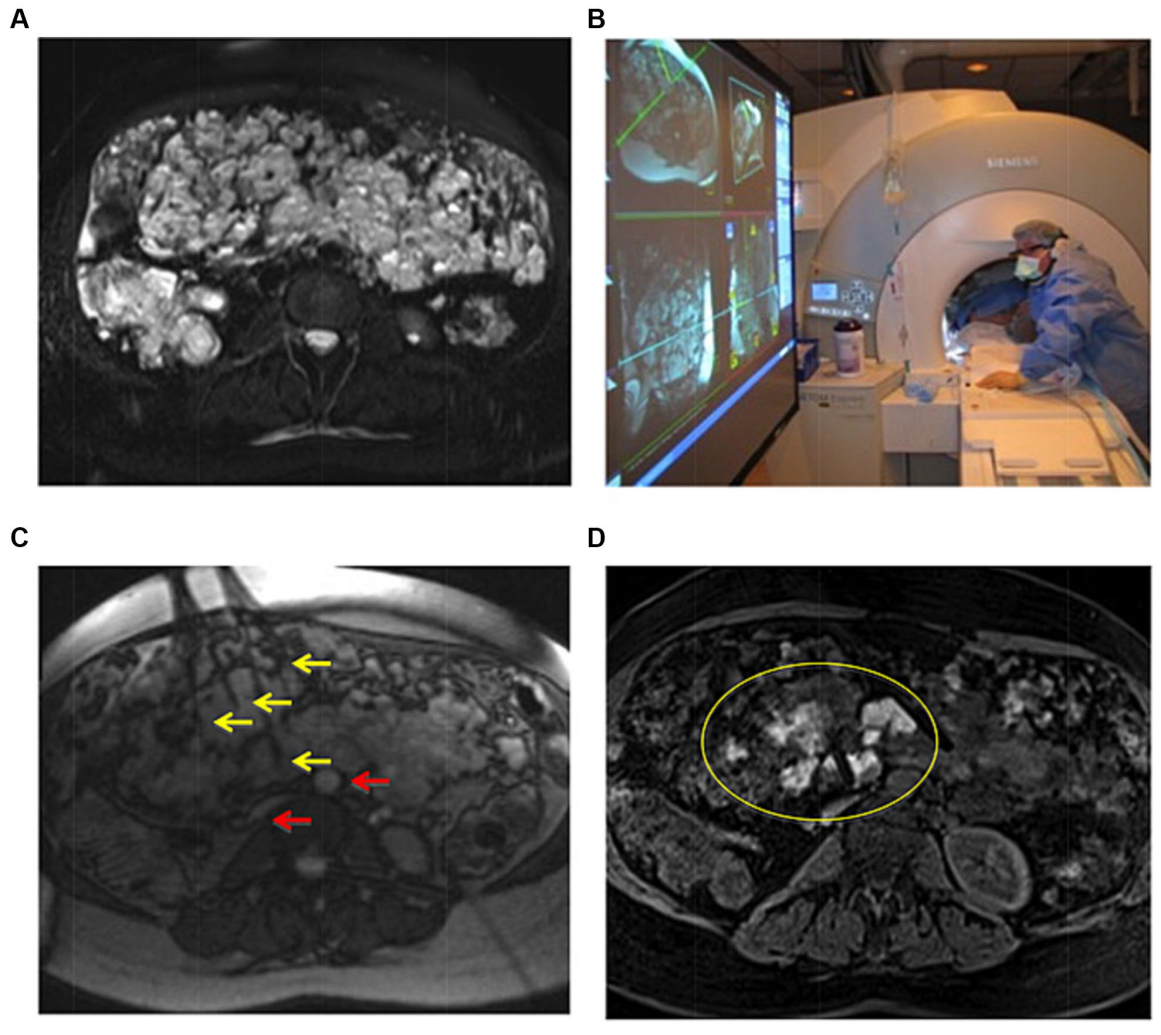
Example of IMRI sclerotherapy application in a patient with complex mesenteric LFVM in the setting of Klippel-Trenaunay syndrome. **(A)** Pretreatment axial T2 TSE with fat saturation demonstrating a massive intra-abdominal VM. **(B)** Scanner setup showing interventional radiologist placing needles under direct MR-guidance using the IFE projected onto a screen in the scanner room. **(C)** Axial RT TrueFISP image showing multiple needles (yellow arrows) placed in deep lesions, avoiding critical structures such as the aorta and vena cava (red arrows). **(D)** Posttreatment axial three-dimensional VIBE confirming delivery of gadolinium-doped sotradecol within multiple portions of the VM. Two of the treatment needles can also be seen (yellow oval). Reproduced with permission, John Wiley and Sons, Inc. (11).

Interventional MRI (IMRI) offers solutions to some of the limitations of ultrasound, fluoroscopy, and CT guidance for LFVM treatment. Again, MRI provides unmatched soft tissue resolution combined with whole-compartment field of view to allow differentiation trans spatial malformations from the adjacent structures. Tissue visualization with MRI is not obscured by bone or air-filled tissue allowing for multiplanar guidance to access deep and perivisceral lesions. Absent of ionizing radiation, IMRI allows for multiple treatment sessions with no added radiation exposure. This provides an advantage when treating certain patient populations with increased susceptibility to radiation making it safe in pregnant and breast feeding women (6). Similar to US guided interventions, MRI guided interventions also provide superior soft tissue resolution when compared to fluoroscopy. Lastly, the overall image acquisition workflow is simplified with all procedural steps performed with a single modality (Figure 1).

First reported uses of IMRI for sclerotherapy occurred between 1999–2004. Initially, this technique relied on a 0.2 T C-arm, open-bore scanners (Magnetom Open, Siemens Medical Solutions, Erlangen, Germany). This system allowed for real-time continuous MR-guidance for needle navigation and sclerosant delivery. However, the system was constrained by limited market availability and comparatively poor image acquisition times and quality (7–9). By 2009, Andreisek et al. opted to overcome the availability and imaging quality barriers with commonly used closed-bore 1.5 T scanners. This setup used projectors connected to graphics outputs which allowed the operator to monitor treatment in real time while the technologist received protocol instructions in the control room (10). By 2016, hybrid 1.5 T MRI-fluoroscopy angiography suites emerged with wide-bore MR scanners; for example, the Magnetom Espree combined with the Axiom Artis X fluoroscopy system and the interactive front end interface (Siemens Healthcare, Erlangen, Germany) (11). These systems were set up in a modular configuration to select fluoroscopic or MR capabilities according to procedural demands (11). Logistics of these procedures in IR such as department configurations that consider surrounding magnetic zones to periprocedural maintenance requirements have been previously outlined and discussed (12). A previously published case example of IMRI guided sclerotherapy is shown in Figure 1. From a health system practice and operational standpoint, dedicated scanners for IMRI procedures are advantageous to avoid compromising daily diagnostic MR volumes. However, the purchasing of such a large capital expense (combined MR/fluoroscopy suites) must be justified by significant case volumes which realistically only occur in tertiary/quaternary academic centers.

## A primer for MRI-guided sclerotherapy of LFVM

### Procedure planning

With the use of T2-weighted Turbo-Spin Echo (TSE) acquisition in combination with fat suppression by spectral adiabatic inversion recovery (SPAIR) and spin echo T1 weighted imaging for regional anatomy, a LFVM can be clearly seen as sharply defined T2 hyperintensities (13, 14). Coil selection is dependent on the location of the lesion and the planned access trajectories; however, a 19 cm linearly polarized circular loop coil which allows a wide area of coverage over a multitude of body surfaces. This coil is readily orientable and can be combined with spine or body phased array coil elements to achieve sufficient image quality for intervention (15).

### Lesion targeting

To access the LFVM, a MR-compatible non-ferromagnetic needle, such as the MReye chromium-cobalt needle (Cook Medical, Bloomington, USA) must be used. Free-hand needle targeting is a common technique used to access lesions along a single standard plane slice. The interventionalist glides the tip of a water-filled syringe while repeatedly imaging the lesion with steady state free procession (FISP) imaging sequences allowing the interventionalist to target the desired skin entry site. Once the syringe tip is positioned, it is pressed to the skin and aspirated to produce a temporary indentation to mark needle entry. The needle is then navigated to the lesion and repositioned until needle tip position is within the desired portion of the lesion. When anatomically feasible to work within the MR bore, navigation can be performed using real-time MR guidance. Needle placement is confirmed once blood return is seen through the needle. A T2-TSE or fast low angle shot (FLASH) acquisition will definitively confirm correct needle position within the LFVM and allow for repositioning and positional confirmation with an injection of dilute gadolinium through the access needle (10).

### Sclerosant delivery

Multiple sclerosants are commonly used for sclerotherapy. Doxycycline (10 mg/cc) is the standard of care sclerosant for LM sclerotherapy (16). Ethanolamine oleate (5%), sodium tetradecyl sulfate (SDS, 3%), and Anhydrous Ethanol (EtOH, 100%) are most commonly used in VM sclerotherapy. Bleomycin, or pingyangmycin, is an antineoplastic antibiotic with antiangiogenic properties which is commonly used to treat VMs that are near critical anatomic structures such as nerves or small compartments like the forearm, wrist, and hand. Unfortunately, Bleomycin and 100% EtOH are incompatible with gadolinium-based contrast therefore a hybrid MR/x-ray fluoroscopy suite is required to monitor delivery in real-time. The effectiveness, dosing regimens, and adverse effects of each agent have been discussed extensively in the literature (17). To visualize the gadolinium compatible sclerosant administration under MR guidance, the sclerosant should be mixed to achieve a gadolinium concentration of 2 μmol/mL. This allows for sclerosant visualization with T2-Half Fourier acquired single-shot turbo spin echo (HASTE) acquisitions or T1-Gradient Reduced Echo (GRE) BEAT IRTTT sequences (6). Careful needle access and sclerosant preparation/delivery technique must be employed to avoid significant air to entry into the sclerosant-gadolinium mixture as air creates imaging artifacts under MR guidance.

### Post-treatment assessment

**Figure 2:**
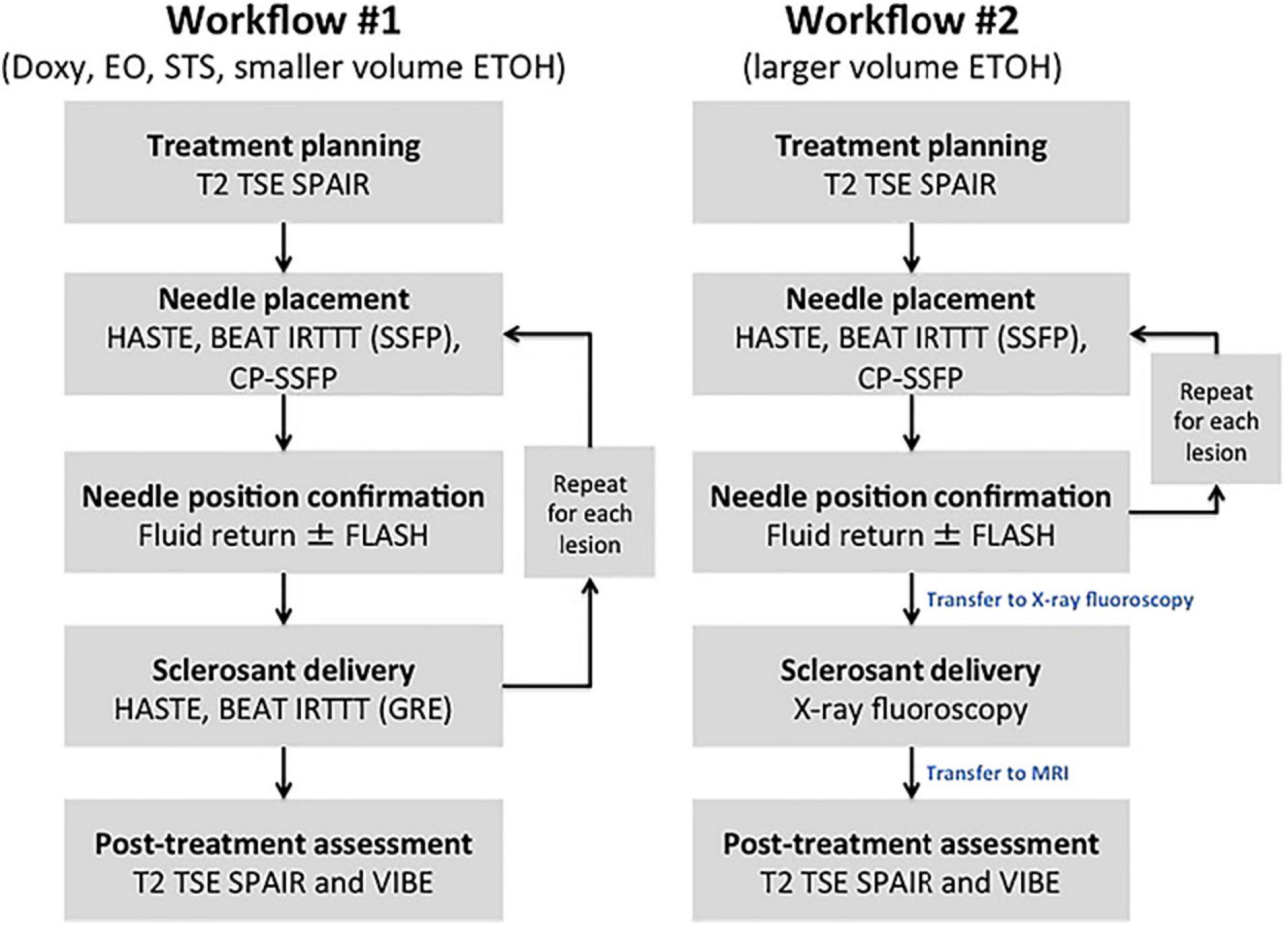
Methods flowchart. Workflow #1 was completed with MR imaging alone; “Milder” sclerosants including Doxy, EO, and STS, as well as smaller volumes of ETOH are delivered with real-time MR guidance. Workflow #2 used the in-suite fluoroscopy, which allowed for the safe delivery of larger volumes of ETOH. Reproduced with permission, John Wiley and Sons, Inc. (11).

To compare pre-and post-treatment response, a T2-TSE-SPAIR acquisition is repeated at the completion of sclerosant delivery. A T1-Fat-supressed volumetric interpolated breath hold examination (VIBE) is also helpful to determine the degree of vascular channel obliteration in high fat content lesions. Previously published sclerotherapy procedure workflows are summarized in Figure 2. Workflow #1 applies to all IMRI sclerotherapy with gadolinium-mixed agents. Workflow #2 assumes hybrid MR/x-ray fluoroscopy suite capabilities for large volume EtOH injections as EtOH is not compatible with gadolinium-based agents.

## Clinical data for MR-guided sclerotherapy

Numerous studies have shown the effectiveness of sclerotherapy for the treatment of venous malformations. In a systematic review, Asdahl et al. evaluated the efficacy of different treatment techniques for sclerotherapy of LFVMs. They found that US and fluoroscopy-guided sclerotherapy for the treatment of LFVMs is efficacious with good to excellent clinical treatment rates of 90–95% in 79.1% (19/24) of studies (18). Given the previously discussed advantages regarding lesion access and visualization, IMRI has been used in patients with lesions that are refractory or not amenable to standard guidance techniques. Lack of access or use of IMRI guidance may account for some of the non-response/unsuccessful treatment rates reported in the literature. Given that this technique does lead to longer procedure times and higher overall cost due to scanner time and equipment it is usually performed on lesions that are not amenable or have failed to respond using standard image guidance techniques.

Summarized in Table 1, several studies have highlighted the efficacy of MR-guided sclerotherapy. In an early trial of MR guided sclerotherapy, Lewin et al. reported the first pilot experience treating maxillofacial LFVMs with IMRI techniques. This trial involved the treatment of 3 patients across 14 procedures using a 0.2 T open-bore scanner. Lesions were targeted by moving a water-filled syringe over the skin surface while rapidly imaging the tip of the syringe until the desired alignment was identified using fast imaging with steady procession (FISP) gradient echo sequence. The lesions were injected with either ethanolamine oleate or SDS 3%. Procedure times were a mean 29 min (range 14–53 min) and LFVMs were consistently reduced in size post-procedure, with improvement in 2 out of 3 cases and resolution of symptoms in 1 out of 3 cases without treatment complications (8). Although conclusions relating to clinical efficacy are limited by sample size, this trial established the feasibility of IMRI sclerotherapy and created an appetite to match the ease of using fluoroscopic guided techniques.

**Table 1:** A brief summary of interventional MRI sclerotherapy studies.

Author, publication year	Study type	Sample size	Intervention	Outcome	Complications
Lewin et al., 1999 (8)	Retrospective review	3 patients with maxillofacial LFVMs across 14 procedures	sclerotherapy	Improvement in 2 out of 3 cases and resolution of symptoms in 1 out of 3 cases	None reported
Boll et al., 2004 (7)	Retrospective review	15 patients with head, neck, trunk, and extremity LFVMs across 76	sclerotherapy	100% technical success rate	None reported
Hayashi et al., 2003 (9)	Retrospective review	13 patients with facial and extremity hemangioma across 14 procedures	sclerotherapy	100% technical success rate	None reported
Andreisek et al., 2009 (10)	Retrospective review	10 patients with extremity LFVMs across 10 procedures	sclerotherapy	100% technical success rate with 60% improvement of symptoms and 30% resolution of symptoms	Rapid drainage of sclerosing agent in 1 patient
O’Mara et al., 2017 (11)	Retrospective review	22 patients across 33 procedures	sclerotherapy	88% technical success rate and 82% improvement of symptoms	3% minor complication rate with 1 patient experiencing self-resolved urinary retention for 24 h
O’Mara et al., 2020 (19)	Retrospective review	6 patients with LFVMs of the neck, chest, and extremities across 10 procedures	sclerotherapy	100% technical success rate and 83% improvement in symptoms	None reported

This early investigation by Lewin et al. paved the way for continued treatment of LFVMs with MR guidance. Boll et al. continued investigation similarly using a 0.2 T open-bore scanner with FISP gradient echo sequence for real-time guidance. This study culminated in outcomes of 15 patients across 76 sclerotherapy procedures in the head and neck, spine, and extremities all treated with ethanolamine oleate. Hayashi et al. used also used a 0.2 T open-bore system to treat 13 facial and extremity hemangioma patients through 14 procedures with MR PSIF ethanolamine oleate. Clinical outcomes reporting was not well-defined limiting overall conclusions or future comparisons, though the authors claimed a technical success rate of 100% of procedures without major treatment-related adverse events (7, 9).

While the use of open-bore scanners laid the initial groundwork for MR guided sclerotherapy, closed-bore systems provide a significant advantage, namely better lesion visualization due to higher field strength, better field homogeneity, and better gradient technology. In a pilot study using a 1.5 T closed-bore system, Andreisek et al. treated 10 extremity LFVM patients in single-session IMRI sclerotherapy with gadolinium-doped 94% EtOH. The group used a commercially available TargoGrid (Daum Medical Devices, Schwerin, Germany) positioned over the skin surface and imaged with 3D-GRE and T2-FSE sequences to determine needle entry sites and navigation. Andreisek et al. reported a 100% technical success rate, however there was a 10% (1/10) adverse event rate: one lesion exhibited rapid regional drainage of sclerosing agent, and, despite application of compression, the patient developed compartment syndrome and subsequently required surgical decompression. Overall, the study reported a mean 56% (range 24–86%) size reduction and reduced T2 signal intensity in LFVMs at 12 weeks post-procedure imaging. Clinical success rates, defined as self-reported improvement or resolution of primary symptoms at 1-year, were 60% (6/10) and 30% (3/10) respectively (10).

In another investigation using a closed-bore scanner, O’Mara et al. used a 1.5 T closed-bore system treating 22 LFVM patients across 33 procedures. The average procedure time was 85 min. The technical success rate, defined as successful blood return after needle placement, followed by sclerotherapy, was 88% (29/33). The clinical success rate, defined as improvement in the patient-reported primary symptom, was 82% (27/33). Notably, regarding the technically unsuccessful procedures, two were performed on lesions which were accessed but ended up being aspirated completely (LM) or were too small to access with the 22G needle. One lesion was intraosseous which could not be accessed with standard MR-compatible needles/equipment. And finally, one lesion revealed a large, brisk draining vein under dynamic intraprocedural imaging which the operators deemed inappropriate to treat with MG-guided sclerotherapy. Procedure-time data from this study suggested that MR-guided sclerotherapy is a skill-based technique with improvement in procedure times with increasing operator experience: the mean difference between the first 7 and last 7 cases was −29 min (first 7: 98 min; last 7: 70 min). There was a minor complication rate of 3% (1/33) with a patient experiencing urinary retention following the treatment of a perirectal VM, however, this resolved after 24 h. No major complications were reported (11).

This same group of investigators continued their work using a 3 T MR system which boasted higher imaging quality with the added benefit of faster scan times. The cohort in this pilot consisted of 6 adults with 10 LFVM lesions in the neck, chest, or extremities. These patients had previous clinically or technically unsuccessful US-guided sclerotherapy. In this study, all procedures were performed with intermittent needle navigation using a T1-and intermediate-weighted TSE sequence with in-and-out technique and injected with gadolinium-doped SDS 3% monitored with T1-TSE subtraction MR fluoroscopy. In this trial, technical success was 100% (10/10) as all lesions were successfully accessed, and clinical success was 83% (5/6) as measured by symptom resolution (4/6) and symptom improvement (1/6) on self-reported symptom questionnaires at a mean of 229 days (range 105–358 days). There were no minor or major complications reported (19). As these patients were previously treated unsuccessfully with US-guided sclerotherapy, this data suggested that MR guided sclerotherapy could be an effective treatment modality for LFVMs that are difficult to treat/failed with traditional US-guidance.

## MR-guided thermal ablation MRI

Several studies have examined the role of thermal ablative techniques for the treatment of refractory LFVMs with multiple case series now reported in the literature. These studies are summarized in Table 2. Thermal ablative techniques, namely laser and cryoablation, are compatible with IMRI sequences described above and a similar workflow can be adapted to each modality. In the setting of challenging lesions which have not responded to percutaneous sclerotherapy or have failed or recurred after surgical resection, thermal ablative techniques offer another treatment option for these patients with LFVMs.

**Table 2:** A brief summary of interventional MRI thermal ablation studies.

Author; publication year	Study type	Sample size	Intervention	Outcome	Complications
Thompson et al., 2015 (23)	Retrospective review	5 patients with lower extremity LFVM	Laser ablation	Complete resolution of symptomatic pain in 100% of patients	20% minor complication rate with 1 patient experiencing self-resolved intramuscular hematoma
Augustine et al., 2023 (20)	Retrospective review	13 patients with cervicofacial LFVM across 22 treatment sessions	Laser Ablation	100% technical success rate and 100% self-reported complete or partial resolution of symptoms	15% minor complication rate with 1 patient experiencing a minor infection which was treated by antibiotics and 1 patient experiencing nontarget thermal injury
Augustine et al., 2021 (25)	Retrospective review	30 patients with extremity and trunk LFVM across 49 laser ablation treatments, 10 cryoablation treatments, and 1 combined treatment	Laser ablation and cryoablation	95% relief in pain and 67% relief in swelling	30% minor complication rate including small hematoma, transient paresthesia, transient weakness, and nontarget thermal injury. None of these required treatment
Autrusseau et al., 2020 (30)	Retrospective review	9 patients with extremity and head and neck LFVM	Cryoablation	Technical success rate of 100% with 67% complete resolution of pain after one session and 100% complete resolution of pain after multiple sessions	None reported
Koepsel et al., 2021 (31)	Retrospective review	5 patients with pedal LFVM	Cryoablation	100% technical success rate with 60% complete resolution of pain after one session and 100% complete resolution of pain after multiple sessions	60% minor complication rate with patients experiencing transient neuropathy which resolved within 6 months
van Breugal et al., 2015 (34)	Case Study	1 Patient with lower extremity LFVM	HIFU	30% lesion volume reduction and self-reported decrease in pain at rest and with exertion	None reported
Ghanouni et al., 2017 (33)	Retrospective review	5 patients with lower extremity LFVM	HIFU	Mean 93% lesion volume reductions and self-reported mean 75% reduction in pain	None reported

## Laser ablation

### A primer for MR guided laser ablation

Laser ablation is a method associated with few complications that can effectively treat certain LFVMs that may be difficult to treat or have failed to respond with sclerotherapy. Patients typically have refractory LFVMs which were not amenable to first line therapies such as surgery or sclerotherapy or they were not viable for such treatments based on the anatomic location of the VM (20). Prior to the procedure, MRI can be used to map the lesion similarly to the pre-procedural process of sclerotherapy.

These procedures can be done under moderate sedation or general anesthesia depending on the location of the lesion, suspected length of procedure, and the patients pain tolerance/co-morbidities. The technique involves the insertion of a laser probe into the LFVM which can be done under real time MR guidance and may involve the assistance of US guidance as well (20). Multiple different types of lasers can be used in the treatment of VMs depending on the size of the lesion. Lesions with small vessels can be treated with pulsed dye lasers, medium sized vessels can be treated with potassium-titanyl-phosphate (KTP) lasers, while large vessels can be treated with neodymium-doped yttrium aluminum garnet (Nd: YAG) lasers. Lasers used to treat VMs with larger vessels typically have a longer pulse duration which is required to cause vessel destruction of these large vessels (21). The laser induces photocoagulation of the blood in the VM which causes the lesion to involute. The operator can vary the number of treatment stages and power used to effectively treat lesions with a wide range of size and severity (22). Furthermore, the treatment pulses can be monitored with proton resonance MR thermometry in real time (23). Due to the adaptability of laser ablation this technique may be more effective in treating large, difficult LFVMs when compared to sclerotherapy (4, 22). Moreover, complications associated with this treatment are rare and typically minor, including scarring, tissue necrosis, and dyspigmentation (25).

### Clinical data

While the laser treatment of LFVMs is a relatively new technique, there are numerous case studies and retrospective studies that suggest that it is effective in treating refractory LFVMs. For example, in a case study from Gocal et al., they discussed the treatment of a large VM on a patient’s tongue which prevented him from closing his mouth. This patient was treated with 3 stages of laser therapy using a Nd: YAG laser with a wavelength of 1,604 nm and power of 15 W. After 3 months’ time, the patient’s lesion had been completely ablated and the patient’s tongue had returned to a normal size (25). Additionally, in a retrospective study of 176 patients with Nd: YAG laser treatment of LFVMs, Poetke et al. reported good to great results in 95% of patients and reported no permanent complications (24). The study by Poetke et al. demonstrates the high success rate of LFVM treatment associated with this technique.

**Figure 3:**
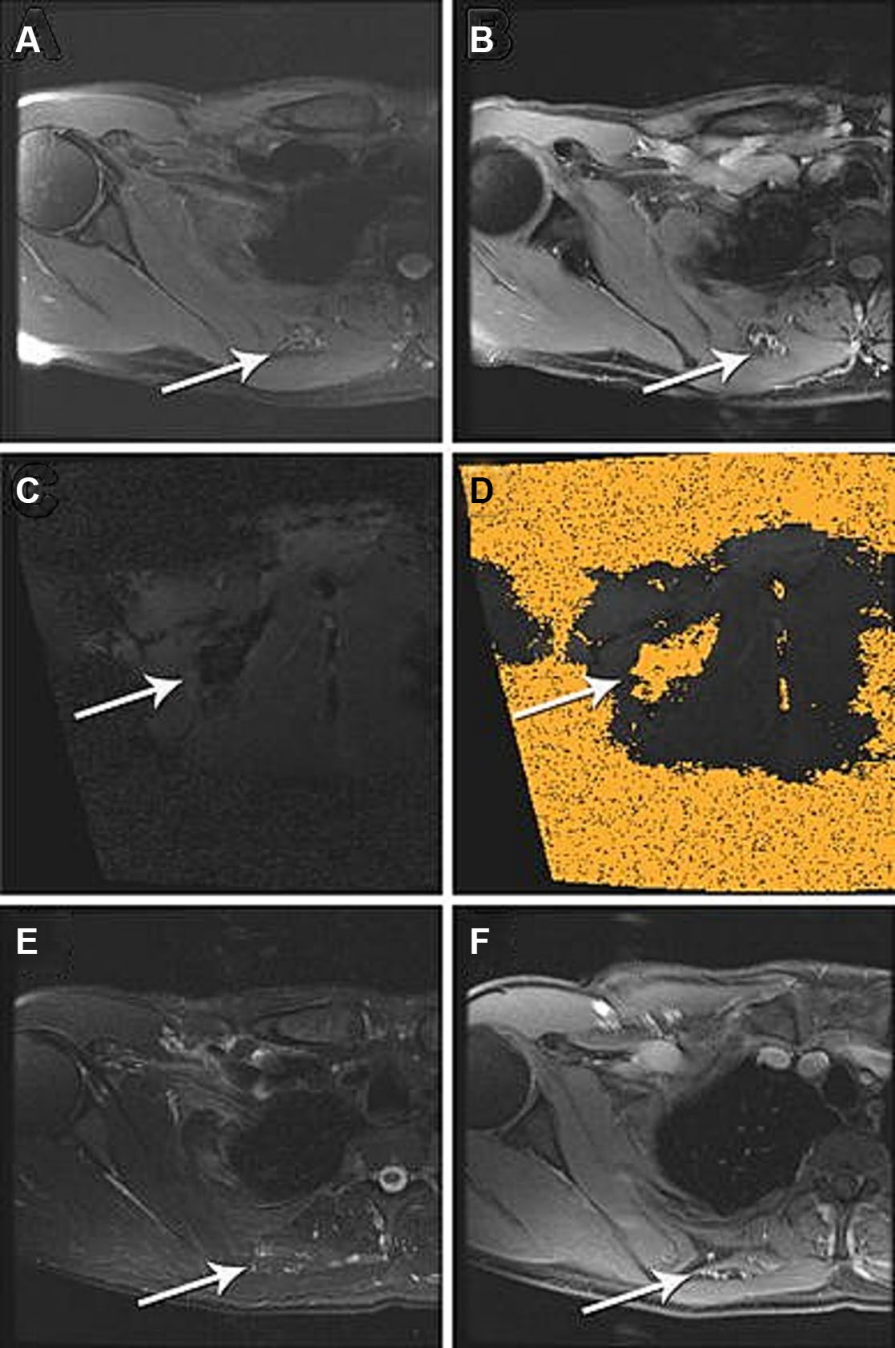
IMRI laser ablation of a LFVM in the soft tissue between the right rhomboid and subscapularis muscles. Pre-ablation **(A)** axial T2-weighted and **(B)** gadolinium-enhanced axial T1-weighted spoiled gradient echo (SPGR) MRI demonstrate heterogeneous abnormal T2 signal [**(A)** white arrow] and contrast enhancement [**(B)** white arrow] within the small vascular anomaly. **(C)** Intra-procedural coronal oblique phase imaging demonstrates real-time tissue heating using proton resonance frequency MR thermometry and **(D)** thermal damage map calculated from this using the Arrhenius equation to estimate the ablation zone. **(E)** One-year post-ablation axial T2-weighted MRI demonstrate no significant T2 signal (white arrow). **(F)** Gadolinium-enhanced axial T1-weight spoiled gradient echo (SPGR) MRI and no enhancement of the vascular anomaly (white arrow). Reproduced with permission from Thompson et al. (23).

This treatment can be effectively combined with MR-guidance to visualize and treat refractory LFVMS. Thompson et al. conducted an early study using MR-guidance paired with thermal ablative techniques to treat lower extremity LFVMs on 5 patients with MR-compatible Visualase laser ablation (MedTronic, Minneapolis, MN, USA). In this study, real time device navigation and deployment guidance was performed using bSSFP sequences in a 1.5 T MAGNETOM Espree (Siemens, Erlangen, Germany). Laser ablation pulses were delivered at a rate of 7 Hz until the lesion was encompassed by a calculated thermal damage map with associated loss of lesion T2 hyperintensity as shown in Figure 3. There was a 20% minor adverse event rate corresponding to a self-resolved intramuscular hematoma. At an average follow-up period of 19.8 months, 100% (5/5) of laser ablation reported complete symptomatic relief of pain (23).

Augustine et al. (20) further published a retrospective report on 13 adult cervicofacial LFVM patients treated with MR-guided laser ablation over 22 treatments. Realtime US-guidance (19/21) or freehand puncture (2/21) were used for titanium introducer placement, then confirmation and laser fiber navigation was performed in either a 1.5 T Magnetom Espree (Siemens, Erlangen, Germany) or Ingenia scanner (Philips Healthcare, Andover, MA, USA). The technical success rate was 100%, and the immediate adverse event rate was 15% (2/13) attributed to self-resolved post-procedure bleeding and surgical site infection, and nontarget ablation presenting as mucosal ulceration which was treated with topical therapy. At a mean follow-up of 8.5 months, 10 patients had an aggregate complete or partial symptom improvement of 100% (10/10) for pain (3/3) and swelling (7/7). Of note, the duration of ablation sessions was reported as a range of 2–4 h which is significantly greater than 1.2–2 h reported for MR-guided sclerotherapy (20).

Lastly, Augustine et al. (25) reported treatment of a cohort of 30 patients with LFVM of the extremities and trunk who underwent 49 IMRI laser ablations, 10 cryoablations, and 1 combination treatment. Of note, 45% (27/60) of these procedures were performed partially with US guidance for device navigation, and real-time MRI for treatment monitoring while 55% (33/60) used MR for device guidance and treatment monitoring including 21 laser ablations employing MR thermometry. By SIR criteria there was a 30% minor complication rate with no reported major complications. Of the 23/27 patients with available follow up at a mean 12.2 months, there was an aggregate partial or complete relief of pain or swelling in 95% (19/20) and 67% (2/3) respectively with mean Visual Analog Scale pain score reductions of −5.7. Lesion size was reported in aggregate for 17/30 patients with available imaging as a reduction in mean maximal dimension of-2.3 cm (pre-treatment 9.5 cm, post-treatment 7 cm). As well, 47% (8/17) had a 76–99% reduction in lesion T2 signal intensity and 58% (10/17) were judged to have a 76–100% reduction in lesion contrast enhancement. This report did not compare the effectiveness of each thermal ablation modality. Regardless, it does suggest that these modalities may be similarly effective at treating moderate-to-severe pain symptoms with only minor self-resolving adverse events (25).

As is seen in these studies, early work on the effectiveness of coupling MR-guidance with laser therapy to treat LFVMs shows promising results in appropriately selected patients.

## Cryoablation

### A primer for MR guided cryoablation

Cryoablation is another tool that appears to be effective for the treatment of patients with LFVMs, especially those with refractory lesions that are not amenable to first-line therapies. Like the aforementioned techniques, the LFVM is mapped using MRI prior to the procedure.

Cryoablation involves the introduction of cryoablation probes into a VM while the patient is typically under moderate sedation or general anesthesia depending on lesion location and patient co-morbidities/pain tolerance. During the procedure, cryoprobes are guided to the target lesion/location which can be monitored under continuous MR imaging by the interventionalist. During cryoablation, super-cooled gas circulates inside the probes, rapidly dropping the temperature in the surrounding tissue leading to apoptosis due to intracellular ice, thereby creating tissue destruction of the LFVM (26). This technique is advantageous with difficult to treat LFVMs because the radiologist can vary the number of cryoprobes inserted into the lesion/target region and the size of the ice-ball depending on the lesion size, shape, and surrounding tissue. Furthermore, hydrodissection can be performed to separate critical surrounding tissues (ureters, bowel, other solid organs etc.) from the site of the lesion thereby preventing damage to normal structures/non-target treatment (27). Two freeze–thaw cycles are commonly used in cryoablation treatment, however the number of cycles, the lengths of the cycles, and the number of cryoprobes inserted can be varied depending on lesion volume, location, and interventionalist’s practice patterns (28, 29). Continuous intraprocedural MR visualization allows the interventionalist to monitor ice ball formation and stop the procedure or ice-ball growth when the entirety of the lesion has been covered or when the ice ball is close to a critical structure (23, 26). While there are risks of nerve injury, hemorrhage, abscess, and nontarget tissue damage, again, these risks can be limited by controlling the size of the ice ball using intraprocedural monitoring. Not only has this method been shown to be effective in treating LFVMs in patients that are not amenable to common first-line treatments such as sclerotherapy (28), but it is also being used in some institutions as a first-line treatment of LFVMs due to its effectiveness and low rate of complications (29). While it is a relatively new technique applied to vascular malformations, it appears to be an exciting and efficacious treatment option for LFVMs.

### Clinical data

As with laser ablation, early research on cryoablation with MR-guidance has shown promise to effectively treat LFVMs. In their systematic review of cryoablation treatment of VMs, Fish et al. reported a weighted mean decrease in VM volume of 92% and a weighted mean decrease in pain score of 77% among 55 VM cases. Fish et al. reported complete resolution of symptoms in 63.6% (35/55) cases and overall improvement in 94.5% (52/55) of cases. Minor side effects of cryoablation included pain, bruising, swelling, and numbness. These side effects were common, however, were transient since they typically resided after two weeks. Two major complications of persistent dysesthesia were reported representing a major complication rate of 3.6% (2/55) (28). While this review did not exclusively assess the efficacy of MR-guided cryoablation, it does demonstrate the effectiveness of treating LFVMs with cryoablation.

Autrusseau et al. reported a retrospective experience treating 9 adult patients with extremity and head and neck LFVM using MR-guided cryoablation. In this study, multiplanar BEAT-IRTT real-time MR fluoroscopy sequences were used to guide IceSeed cryoprobes (Galil Medical/Boston Scientific, Marlborough, MA, USA) in real time. A mean of 3.7 cryoprobes and mean 2.4 freezing cycles were required for treatment to cover the whole lesion. The authors reported a technical success rate of 100 and 0% adverse events rate. At a mean follow-up time of 548 days, 67% (6/9) patients reported complete resolution of pain without recurrence after single session. The remaining 3 patients underwent further cryoablation treatments, after which they reported complete resolution of symptoms at post-procedure follow-up (range 25–153 days) (30). Koepsel et al. reported another experience treating 5 foot LFVM patients with MR-guided cryoablation in a 1.5 T Philips Ingenia system (Philips Healthcare, Andover, MA, USA) with IceRod cryoprobes (Galil Medical/Boston Scientific, Marlborough, MA). Continuous proton-density weighted TSE was used for real time monitoring of a median 3 needles during cryotherapy. The technical success rate of this series was 100%. 60% (3/5) of patients had a minor complication, namely transient neuropathy which resolved by 6 months post-procedure. There was complete resolution of pain in 60% (3/5) of the patients after one session, and the remaining 2 patients underwent repeat cryoablation to achieve full resolution of pain (31).

Current studies are limited by their retrospective design and or small sample sizes with heterogenous reporting of clinical outcomes. Nevertheless, the studies reporting on cryoablation as well as laser ablation using IMRI present promising and efficacious new treatment options for LFVMs.

## High-intensity focused ultrasound (HIFU)

### A primer for HIFU

High-intensity focused ultrasound is the quintessential IMRI thermal ablation technology which allows for truly non-invasive thermal ablations with real-time therapy monitoring via MR-thermometry (32). As previously discussed, the LFVM is mapped prior to the procedure using MRI and the patient is typically placed under moderate sedation or general anesthesia for the duration of the procedure.

During the procedure, the target tissue is exposed to high intensity ultrasound causing tissue ablation in the target region. This procedure typically begins with low energy sonification to calibrate the device followed by HIFU treatment with varying sonification duration and energy in order to achieve the desired increase in temperature causing tissue ablation of the VM. Procedure time is variable depending on the size of the malformation and the US parameters selected (33). During the procedure, MR-thermometry can be used to monitor the heating to the target region/lesion and the surrounding region which allows the interventionalist to monitor the temperature of the tissue as well as to avoid surrounding critical structures such as other nerves or vessels (34). HIFU provides a unique advantage to treating LFVMs in that it is a non-invasive technique that can be monitored in real-time. While the data surrounding LFVM treatment with HIFU are limited, early investigations indicate that HIFU may be an effective, non-invasive treatment method for LFVM.

### Clinical data

**Figure 4:**
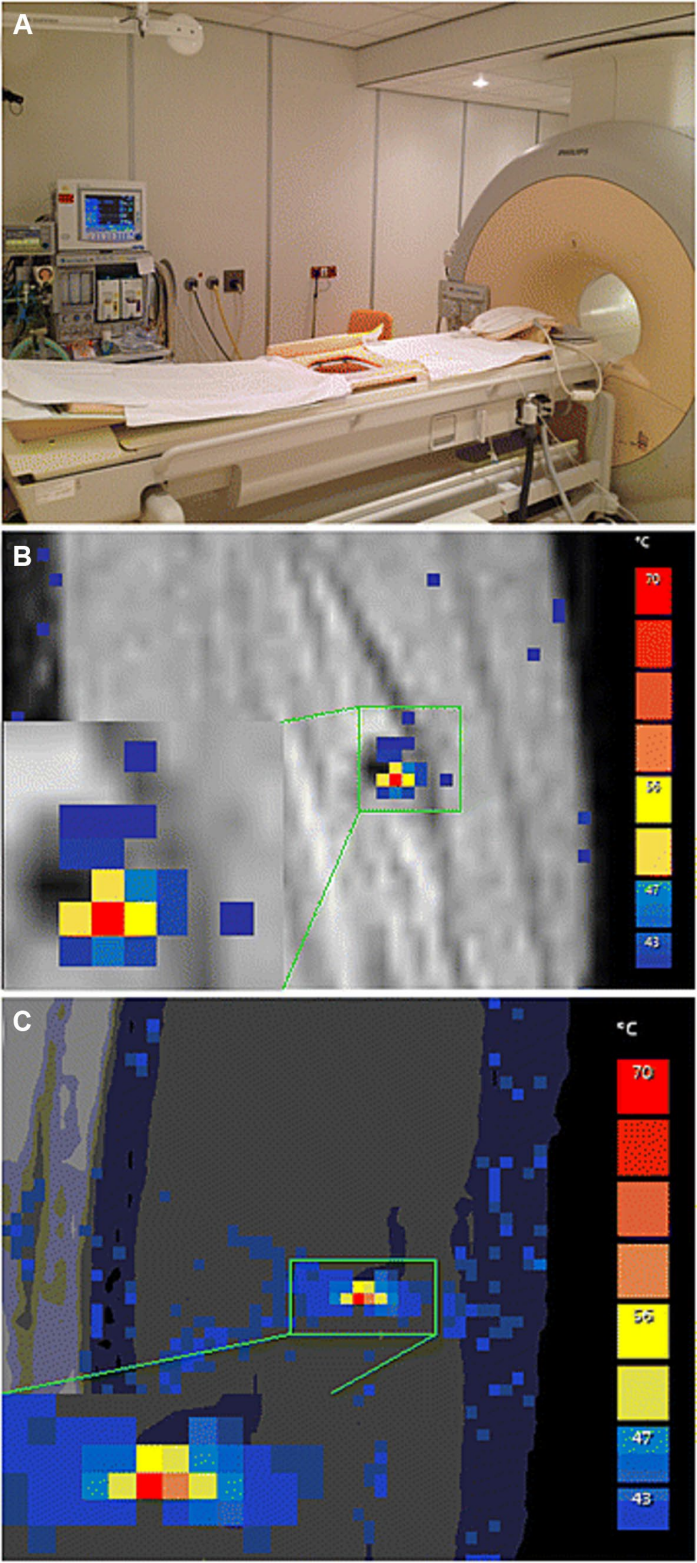
**(A)** Philips Sonalleve IMRI-HIFU device setup including integrated active skin cooling pad. **(B)** Coronal and **(C)** sagittal planes of MR-Thermomentry during sonication delivery. Reproduced under creative commons license CC-BY, Springer Nature (34).

Van Breugel et al. reported a case of MR-guided HIFU (MR-HIFU) applied to a painful intramuscular anterior tibialis venous malformation in an adult male which was over 2 mm of the skin and the posterior tibial neurovascular bundle (34). A single-session of MR-HIFU was performed in a 1.5 T Sonalleve system (Philips Healthcare, Andover, MA, USA) applying point sonication to reach lesion temperatures of 62–81°C during a 45-min treatment session (demonstrated in Figure 4). Immediate post-procedure MR gadolinium-enhanced multiplanar reconstructed T1-weighted high resolution isotropic volume examination (MPR-THRIVE) was performed to confirm absent contrast filling of the vascular lesion. At 3 months post-procedure, MR short t-inversion recovery (STIR) imaging revealed 30% volume reduction (1.9 mL pre-procedure-1.3 mL post-procedure) of the treated portion of the lesion. Self-reported pain on an 11-point scale decreased from 8 with mild exertion pre-procedure to 0 at rest and < 2 with exertion at 1 month and sustained through 13 months post-procedure (34).

Consistent with the findings of this case report, Ghanouni et al. retrospectively analyzed the treatment of 5 adult male patients with lower extremity LFVM treated with MR-HIFU. MR-HIFU was performed in the ExAblate 2100 system (InSightec, Tirat Carmel, Israel) delivering spot sonication to reach average lesion temperatures of 55.3°C (range 47.3–78.0°C) during a median 119 min of treatment (range 50–202 min). The median follow-up duration was 9 months (range 4–36 months). There was a median 93% lesion enhancing volume reductions (pre-treatment 8.2 mL, post-treatment 0 mL) measured by multiplanar FSE and GRE MRI. In addition, there was also a 75% reduction (pre-treatment 8.4, post-treatment 1.6) in the mean 11-point pain scale scores. Finally, there were no procedure-related complications reported in this series (33).

These studies laid a foundational framework that demonstrate the promise of MR-HIFU ablation to treat LFVM using an MR-guided, completely non-invasive technique. Despite that, MR-HIFU has significant shortcomings. First, lesions near air-filled structures such as the airways or any portion of the alimentary tract or that are near bone may be incompatible with MR-HIFU as air and bone will scatter the focused US beam. Second, MR-HIFU procedures can be long, depending on the size of the lesions. The procedures in the Ghanouni series were in the order of two hours per session, markedly longer than sclerotherapy sessions and comparable to other ablative technique. Though not explicitly studied, these longer procedure times will likely limit adoption given the cost of MR scanner time and equipment relative to US and fluoroscopy guided sclerotherapy.

## Complimentary MR-guided technologies

One of the challenges associated with MRI guided needle-based therapies is the amount of time required for needle navigation. This is an issue not only for sclerotherapy, but also for laser ablation and cryoablation where long procedural times require multiple treatment sessions for a LFVM. Multiple examples of hardware-assisted needle navigation exist in the literature, including rigid robotics and augmented reality (35–38). However, there is a high skill ceiling of operator training required with MRI, thus a supplementary technology must balance solving specific workflow challenges with further operational barriers and ergonomic convolution.

Closed bore systems provide the advantage of superior image quality, but they also present the difficulty of patient access and needle navigation during the procedure. Namely, the operator can have a difficult time working within the bore limiting the rapidity of access. Augmented reality assistance for needle navigation can mitigate challenges in patient access in a closed bore system as well as decrease the procedure time (39). In a 2018 study, Mewes et al. offered a possible solution to these challenges through the use of augmented reality guided needle placements. In this study, the group created a 2-dimensional and 3-dimensional augmented reality visualization system that assisted the interventionalist in needle placement. In a phantom body experiment, the group found the 2-dimensional and 3-dimensional systems to be equally accurate with target distance errors of 2.0 ± 0.6 mm and 1.7 ± 0.5 mm. Furthermore, the group found no significant difference in errors between a group of interventionists and a group of inexperienced users with a technical medical background suggesting the systems are easy to learn, use, and adapt to (39). Augmented reality guided MR needle interventions need to be tested specifically with VM treatment for efficacy and efficiency, but we postulate that this technology may prove beneficial to overcoming the difficulties of patient access with the use of closed bore MRI systems while maintaining accuracy of needle interventions when targeting LFVMs in the future.

**Figure 5:**
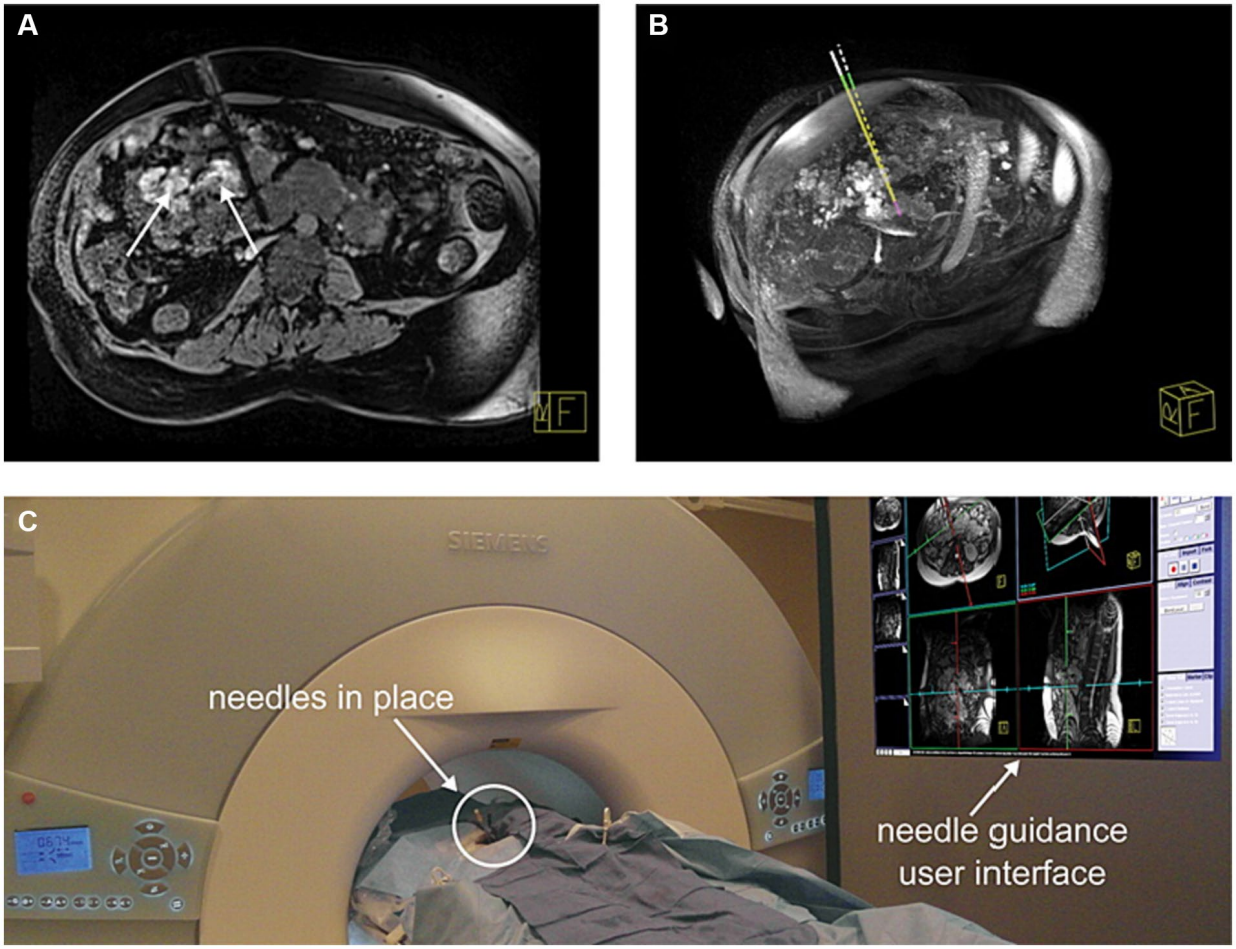
Successful needle placements (four needles) for sclerotherapy of a complex intraperitoneal venous malformation (VM) in a 40-year-old adult with Klippel–Trenaunay syndrome who could not be treated by ultrasound and x-ray fluoroscopy-guided treatment. **(A)** Verification dataset of needle placement into a VM adjacent to vena cava and other critical structures. Areas of the VM treated earlier in this procedure with gadolinium DTPA-doped SDS 3% astill show enhancement in the image (arrows). **(B)** Comparison between planned (dashed line) and actual needle trajectory. **(C)** Patient in the scanner with needle guidance user interface displayed on a screen in the MR scanner room. Reproduced with permission, John Wiley and Sons, Inc. (40).

Another challenge associated with IMRI guided needle-based therapies is related to the amount of procedural time required for needle navigation. Due to its iterative nature, the in-and-out approach to placing the needle into the malformation consists of most of the active procedure time. Furthermore, not all lesions are accessible via single 2D slice trajectories and may require complex orthogonal approaches. A scanner-integrated semiautomatic software package to plan and guide needle trajectory was developed by Rothgang et al. (40). From an initial high-spatial resolution MR dataset in the procedure planning phase, multiplanar reformatting, maximum intensity projection, and volume rendering are performed to characterize the lesion. Using two mouse clicks, the operator selects entry and target points. Needle trajectory is proposed by an algorithm which is then reviewed by the operator to ensure avoidance of critical neurovascular and visceral structures, as shown in Figure 5. The planned trajectory is overlayed such that the operator may adjust actual needle placement throughout the procedure. In a prospective trial by O’Mara et al. employing this semiautomated software planning and targeting technique in a 1.5 T system, treating 22 LFVM patients across 33 procedures resulted in mean decreases in targeting time and procedure time of 25 min and 28 min, respectively (10). Along with sclerotherapy, these studies also provide promising early results for the use of this software package to assist in the placement of cryoprobes and laser probes as these thermal ablation techniques are typically associated with long procedural times which may require multiple treatment sessions (25).

MR thermometry is another recent innovation that has been adopted to assist in both MR guided HIFU and MR guided laser ablation (20, 33, 34). When MR thermometry is employed in conjunction with these techniques, it allows the radiologist to monitor tissue thermal distribution in real time which can help prevent damage to the tissue surrounding the target lesion (41). MR thermometry also allows the radiologist to monitor the temperature of the target tissue during the ablation procedures which ensures that sufficient temperatures are reached during the procedure (34).

**Figure 6:**
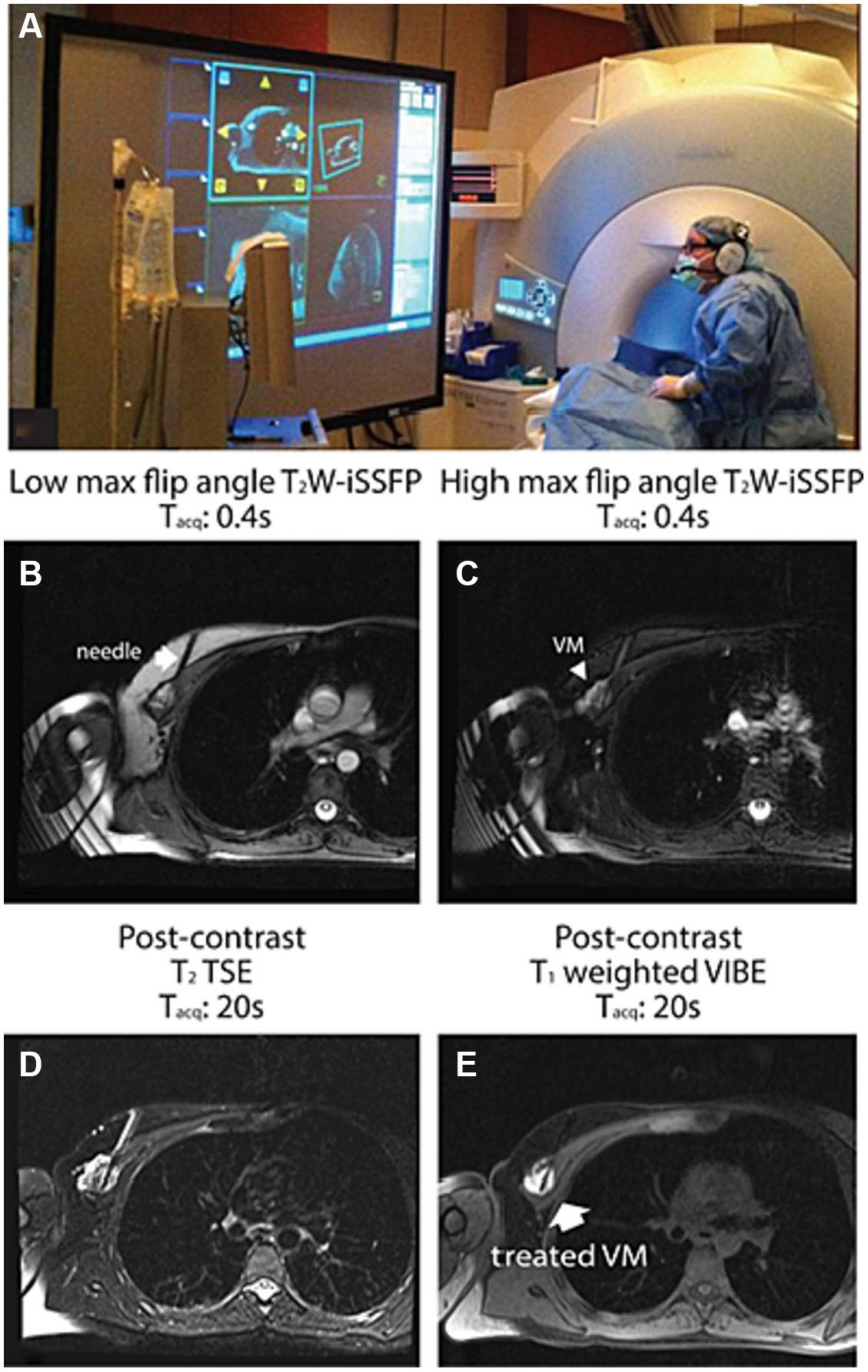
An example of a MR-guided sclerotherapy using T2W-iSSFP. This patient underwent treatment for a deep chest wall VM which was not accessible by US guidance. **(A)** Real-time MR video was integrated into an interventional guidance visualization software and displayed on an in-room projector. **(B)** High and **(C)** low max flip angle real-time T2W-iSSFP images sampled during needle insertion. Real-time T2W-iSSFP offers heavy T2-weighting and high image quality to visualize the lesion and its extent, as well as the surrounding critical structures such as the normal blood vessels and the lung. Low max flip angle T2W-iSSFP emphasizes the depiction of the needle [the arrow in **(B)**] and the surrounding soft tissue. High max flip angle T2W-iSSFP provides heavily T2-weighting for visualizing the VM emphatically [arrowhead in **(C)**]. **(D)** Post-contrast T2-TSE and **(E)** post-contrast T1-weighted VIBE 20 demonstrate the success of the needle placement and the filling of the sclerosant (sodium tetradecyl sulfate). Reproduced with permission, John Wiley and Sons, Inc. (42).

Another important imaging development for the advancement of MR guided treatment of LFVMs is T2-weighted interrupted steady state free procession (T2-iSSFP) sequence. Commonly used sequences for diagnosis, needle navigation, and sclerosant delivery have distinct advantages, and disadvantages which render them suboptimal in other procedural steps. T2-TSE-SPAIR acquisitions are too slow for real-time needle guidance. Faster acquisitions, such as real-time HASTE and T2-bSSFP produce images with blurry edges or poor T2 contrast, respectively. A novel readily implemented MR sequence was developed which integrated the time-resolution advantages of T2-HASTE and T2-bSSFP while flexibly maximizing tissue resolution. T2-iSSFP is a variable flip angle bSSFP sequence which has been previously described. T2-iSSFP allows the operator to adjust the degree of T2 contrast reactively to identify tissues surrounding the needle tip during navigation by only manipulating the maximum flip angle parameter. Procedural examples of T2-iSSFP implementation are included in Figure 6 (42).

The studies presented provide insight into some potential technological advances in needle navigation and use of intraprocedural MRI for sclerotherapy, cryoablation, laser ablation, and HIFU. While the possible applications of needle guided technology is exciting, further research is required to assess the feasibility of these navigation technologies specifically for the treatment of LFVMs. To explore the applicability of these techniques, future research should focus on these technologies exclusively applied to treatment of LFVMs using iMRI and should include a more robust sample size.

## Discussion

The present studies suggest that MR guided intervention is a valuable resource for the interventionalist when treating LFVMs with a variety of methods. Treatment of LFVMs has historically relied heavily on the use of US, CT, or X-ray fluoroscopy guidance. Intraprocedural MRI, however, provides advantages in that a wider range of LFVMs in different anatomical locations among a diverse range of patients can be treated with intraprocedural MRI guidance. Sclerotherapy was quickly adopted as a common first-line LFVM treatment using MRI guidance and showed success in treating LFVMs as is evident in the studies presented. Nevertheless, the studies suggest that thermal ablation techniques, namely laser ablation, cryoablation, and HIFU, are better employed for the treatment of certain refractory LFVMs not amenable to sclerotherapy. While the use of thermal ablation techniques in conjunction with MR guidance is not yet as well researched, these techniques may be an exciting step forward in the future treatment of refractory LFVMs not amenable to common first-line therapies.

There are numerous challenges that must be overcome when adopting MR guided interventions in a new setting. First, the setup of an IMRI suite requires careful planning to allow for different procedures such as cryoablation and laser ablation. Tools, approved by the Food and Drug Administration, have already been developed for HIFU, cryoablation, and laser ablation which are compatible with IMRI, making these procedures feasible under MR guidance (43). As discussed by Stefanini and Simonetti in their article on IMRI suite setup, there are numerous considerations to consider when setting up such a suite. First, the suite must be larger in size to allow for patient access and machinery and software required for different ablation procedures with real time monitoring. Second, a separate room must be organized for storage of gasses used for cryoablation (44). Another challenge associated with IMRI treatment of LFVMs is associated with the cost due to scanner time and equipment. The introduction of technologies such as augmented reality or software assisted needle targeting under MR guidance has the possibility of decreasing procedure times significantly thereby limiting the cost of these procedures. Nevertheless, further research is required to investigate the application of these technologies to the treatment of LFVMs.

While there are challenges in the setup of IMRI suites and the costs associated with these procedures, MR guided interventions have numerous exciting clinical implications for the treatment of LFVMs. First, intraprocedural MRI provides superior soft tissue image quality allowing for better targeting and treatment of large, complex LFVMs or LFVMs with proximal nerves or tissues susceptible to damage. Furthermore, thermal ablation techniques, such as cryoablation, laser ablation, and HIFU, combined with intraprocedural MRI can be an effective tool for treating LFVMs that were not amenable to prior treatment and they are associated with a low complication rate. Lastly, MR guided interventions do not expose patients to increased doses of ionizing radiation allowing for safe treatment of patients with LFVMs and for multiple treatment sessions given complex malformations. Although there are challenges associated with the adoption of MR guided interventions, the literature suggests that it is a critical tool in the successful treatment of certain LFVMs, especially those not amenable to other treatments.

## Conclusion

While MR guided interventions are limited by longer procedural times, higher cost due to scanner time and equipment, and the need for a tertiary or quaternary levels of care, they still offer an opportunity for interventionalist to treat patients with LFVM safely and effectively in a radiation-free environment. As new technologies such as laser ablation, cryoablation, and HIFU gain more adoption for the treatment of LFVM, they can be used alongside sclerotherapy to improve clinical outcomes for both adult and pediatric patients.

## Author contributions

CB: Conceptualization, Investigation, Writing – original draft, Writing – review & editing. DH: Conceptualization, Data curation, Investigation, Writing – original draft, Writing – review & editing. NN: Conceptualization, Data curation, Investigation, Writing – original draft, Writing – review & editing. CW: Conceptualization, Data curation, Investigation, Supervision, Writing – original draft, Writing – review & editing.
